# Facebook Experiences of Users With Traumatic Brain Injury: A Think-Aloud Study

**DOI:** 10.2196/39984

**Published:** 2022-12-16

**Authors:** Reihaneh Ahmadi, Hajin Lim, Bilge Mutlu, Melissa Duff, Catalina Toma, Lyn Turkstra

**Affiliations:** 1 Department of Psychology, Neuroscience & Behaviour McMaster University Hamilton, ON Canada; 2 Department of Communication Seoul National University Seoul Republic of Korea; 3 Department of Computer Sciences University of Wisconsin-Madison Madison, WI United States; 4 Department of Hearing & Speech Sciences Vanderbilt University Medical Center Nashville, TN United States; 5 Department of Communication Arts University of Wisconsin-Madison Madison, WI United States; 6 School of Rehabilitation Science McMaster University Hamilton, ON Canada

**Keywords:** traumatic brain injury, rehabilitation, disability, cognitive communication, social media

## Abstract

**Background:**

A critical gap in our knowledge about social media is whether we can alleviate accessibility barriers and challenges for individuals with traumatic brain injury (TBI), to improve their social participation and health. To do this, we need real-time information about these barriers and challenges, to design appropriate aids.

**Objective:**

The aim of this study was to characterize the ways people with TBI accessed and used social media websites and understand unique challenges they faced.

**Methods:**

We invited 8 adults with moderate to severe TBI to log onto their own Facebook page and use it as they regularly would while *thinking aloud*. Their comments were recorded and transcribed for qualitative analysis. We first analyzed participants’ utterances using a priori coding based on a framework proposed by Meshi et al to classify adults’ motives for accessing social media. We next used an open coding method to understand the *challenges* that people with TBI faced while using Facebook. In other words, we analyzed participants’ needs for using Facebook and then identified Facebook features that made it challenging for them to meet those needs.

**Results:**

Participants used all categories of codes in the framework by Meshi et al and provided detailed feedback about the Facebook user interface. A priori coding revealed 2 dimensions that characterized participants’ Facebook use: whether they were active or passive about posting and self-disclosure on Facebook and their familiarity and fluency in using Facebook. The open coding analysis revealed 6 types of challenges reported by participants with TBI, including difficulty with language production and interpretation, attention and information overload, perceptions of negativity and emotional contagion, insufficient guidance to use Facebook, concerns about web-based scams and frauds, and general accessibility concerns.

**Conclusions:**

Results showed that individuals with TBI used Facebook for the same reasons typical adults do, suggesting that it can help increase social communication and reduce isolation and loneliness. Participants also identified barriers, and we propose modifications that could improve access for individuals with brain injury. On the basis of identified functions and challenges, we conclude by proposing design ideas for social media support tools that can promote more active use of social media sites by adults with TBI.

## Introduction

### Traumatic Brain Injury and Social Participation

Social isolation is a common reality for many people with traumatic brain injury (TBI) [1,2], especially when the injury is severe enough to affect thinking and behavior. TBI-related social isolation has profound negative effects on well-being [3] and is associated with psychological symptoms such as anxiety and depression [4]. Social inclusion has the opposite effect: adults with TBI who have greater social engagement and community integration report greater life satisfaction and less emotional distress [5,6]. Social connection also plays a critical role in regaining identity after a TBI [7], which in turn is central to injury recovery [8].

Following a TBI, an individual must overcome many cognitive, physical, and psychological barriers, making it difficult for them to return to their everyday social lives [9]. Cognitive impairments in particular can cause behavioral and communication changes that are difficult for friends and family members to understand [9]. These impairments include memory problems, slower thinking speed, difficulty recognizing social cues across multiple types of media, and impairments in higher-level executive functions such as reasoning as well as behavioral and psychosocial changes and social inappropriateness [10-19]. These cognitive impairments translate into communication challenges, especially in everyday social interactions [5,16,20], and are thought to be a main contributor to loss of friendships and social isolation in the chronic phase after injury [21].

### TBI and Social Media

Social media platforms may offer a mechanism for adults with TBI to increase their social connections. These platforms can provide opportunities to maintain preinjury networks and make new connections with a broad community of users. A user community can include groups with shared interests and groups with shared experience, who may be a source of support and encouragement for people with TBI, as well as groups that advocate and raise awareness about TBI. All of these provide opportunities for meaningful social interactions after injury. As noted by Brunner et al [22], regaining participation on social media platforms can be a meaningful goal for a person with TBI, and goals with personal meaning are more likely to be achieved in rehabilitation. Most platforms also provide mechanisms for asynchronous communication, a significant benefit for individuals with TBI who have difficulty understanding and producing social language under time pressure [23,24]. In adults without TBI, use of social media has been shown to provide many psychological and relational benefits, including increased social connectedness and sense of belonging, and decreased loneliness for users [25,26]; as well as opportunities for relationship maintenance [27,28]. These benefits map directly to the needs of people with TBI.

There is evidence that people with TBI want to be engaged in social media but encounter significant barriers with typical platforms that were not designed to accommodate users with cognitive impairments [1,3,29,30]. The main barriers relate to the cognitive demands of accessing and using social media, including the need to remember how to access a site, find relevant information and ignore distractions, and understand stated and implied content, and do all of these things quickly and efficiently [30]. Social communication impairments may also prevent individuals with TBI from being able to engage appropriately with others on social media or understand content they encounter. These factors may generate a negative experience for individuals with TBI and discourage them from using social media in the future.

### This Study

For individuals with TBI to benefit from social media use, it is critical to address the barriers they may encounter so we can provide solutions to overcoming those barriers. Studies to date have advanced our understanding of the needs and challenges of adults with TBI but are largely based on retrospective reflection (eg, via self-report surveys) or analysis of previous posts [29,31-33]. Retrospective recall and self-reflection can be particularly challenging for adults with TBI, who commonly have impairments in declarative recall and metacognition [34,35], and analysis of posts does not illuminate the process of accessing social media in real time.

To overcome challenges of retrospection-based methods, we used a *think-aloud* method to collect responses of adults with TBI while accessing their social media. Think-aloud methods ask participants to verbalize their thoughts and actions as they navigate and use an interface [36]. Think aloud has been widely used as a technique to understand users’ system use patterns and identify usability issues in a wide variety of user interfaces [37,38]. Think aloud has been recognized as a valid tool to both collect reliable descriptions of user behaviors and also naturally encourage users to describe motivation, doubt, confusion, and challenges in using computer interfaces [37,38].

The aim of this study was to use the think-aloud method to characterize the ways people with TBI accessed and used social media websites, including challenges they faced. We focused on Facebook because it is the most popular social networking site worldwide and the dominant social media platform for adults, with 2.8 billion monthly active users, 1.84 billion of whom log on on a daily basis [39]. In our recent survey of 50 adults with TBI, Facebook also was the most commonly used platform [33]. Facebook provides a mechanism for users to build and maintain a wide range of social connections, from family, friends, colleagues, and coworkers to strangers from around the world. Facebook allows users to share messages and images with their connections and involve themselves in local events, pages they support, or groups; thus, it may be particularly useful for people with TBI as they rebuild identity and interests after injury.

## Methods

### Recruitment

Participants were 5 females and 3 males in the chronic stage after moderate to severe TBI. All participants were recruited from individuals and agencies providing services to adults with TBI in the local area in Southern Ontario, in Canada. Organizations providing services to individuals with TBI were initially contacted via email and invited to share study information with adults with TBI. If an adult with TBI expressed interest in the study either they were invited to visit the university campus to participate in the study, or the principal investigator would travel to the participant’s location. Participants’ ages were from 26 to 64 years and the mean age of participants was 49 (SD 14.69) years. The mean years of education were 12.3 (SD 2.06) years.

Participants met the following inclusion criteria: (1) moderate to severe TBI, defined according to standard injury criteria [40], that is, loss of consciousness for 30 minutes or more, posttraumatic amnesia of 24 hours or more, and worst Glasgow Coma Scale full score in the first 24 hours of less than 13, or 13 or higher with evidence of brain damage; (2) more than 6 months post injury, as social impairments emerge in the chronic stage after injury; (3) self-identification as a native English speaker, to rule out challenges and barriers related to English proficiency; (4) aged 18 to 65 years, as individuals in this age group are the highest Facebook users [41] and to limit potential confounds due to cognitive decline with age after TBI [42]; and (5) self-reported active social media use or passive social media use (eg, observing but not posting). Average scores on neuropsychological tests were consistent with larger studies of adults with TBI [43], that is, showing impairments in task switching, processing speed, and delayed recall of verbal information

### Informed Consent and Study Intake Form

Participants were provided with a consent form outlining the purpose and procedure of the study and risks and benefits. As users would be accessing their own Facebook accounts, the risks and benefits section of the consent form included specific language about Facebook’s privacy policy. The first author then completed a study intake form in collaboration with each participant. The intake form consisted of questions about participants’ age, sex, race, years of education, and TBI history.

### Measures to Characterize the Sample

The Common Data Elements Committee for TBI research [44] recommended standardized tests that participants with TBI should complete, to characterize the sample and allow researchers to compare results obtained by different studies and publications. Per the Common Data Elements Committee recommendations, participants completed the following tests: the California Verbal Learning Test [45], Wechsler Adult Intelligence Scales Processing Speed Index tests [46], and Trails making tests A and B (Trails A and B) [47].

### Think-Aloud Procedure

Participants were asked to log into their personal Facebook account on the laboratory computer and use it as they typically would while *thinking aloud*. Camtasia software [48] was used to capture a screen recording of each participants’ activity while they were using their social media accounts and a video camera was physically placed in front of the participant to capture their facial expressions and body movements. The first study participant was instructed to *think aloud* while using their Facebook account for 60 minutes. If the researcher noticed that the participant was not *thinking aloud* for a period longer than 60 seconds, they would provide the participant with a reminder to do so.

On the basis of feedback from the first participant, remaining participants were instructed to use their personal Facebook account for 15 minutes while they were *thinking aloud*. We also observed that some participants had challenges simultaneously talking and accessing their Facebook pages, so we generated a list of prompt questions to use if participants did not speak while accessing their Facebook account. Prompts included the following: “Are you more comfortable socializing online or in person?”; “When you come across a public post on Facebook, are you likely to comment with other users in the comment section?”; “Who do you socialize and interact with on Facebook?”; “Are you actively posting content on your Facebook profile?”; “If you could filter out content that you see on Facebook, what would you filter out?”; “What do you think of the Facebook format?”; “Do you find it easy to use the platform?”; and “What would you change about the format?”

### Ethics Approval

All documents and procedures were approved by the Hamilton Integrated Ethics Review Board (Study No. 4974).

### Scoring and Data Analysis

Comments were recorded, transcribed, and segmented into turns for analysis. Turns were defined according to criteria summarized by Traum and Heeman [49] as speech by a single speaker that was syntactically complete, defined a single speech act, was an intonational phrase, and was separated by a pause. Utterances were entered into Atlas.ti [50] for analysis.

### A Priori Coding

Meshi et al [51] described social media use according to a biopsychosocial framework. They proposed that social media is a platform for users to fulfill their basic human need to connect and “manage their reputation,” which ultimately would result in greater well-being and enhance social connections that would promote reproductive success. The authors identified five key behaviors adults used to meet their needs for social connection and reputation management: (1) broadcasting information, including words and images that are personal or shared from others; (2) observing others’ broadcasts; (3) giving feedback on others’ broadcasts, for example, via *liking* posts; (4) receiving feedback on broadcasted information; and (5) engaging in social comparison, which can be by comparing posts and feedback or using metrics such as network size or relationship status. Meshi et al [51] linked these 5 uses to 3 human cognitive functions: social cognition (also known as social thinking or *mentalizing*), self-referential cognition, and social reward processing. Each cognitive function was mapped to specific neural networks, based on human and animal research in neuroscience. The Meshi et al [51] framework was intended to guide researchers in using social media to study brain functions. It worked equally well, however, to describe social media use in adults with TBI, who often have impairments in the 3 cognitive domains listed.

We chose the Meshi et al [51] framework for this study because it focused on use of social media to satisfy basic social needs, and so provided a way or conceptualize Facebook functions for our participants. We used the 5 key behaviors described by Meshi et al [51] to create a high-level coding taxonomy. As one aim of the study was to obtain feedback specifically about Facebook, we added a coding category for Facebook feedback related to the user interface. Furthermore, 2 researchers coded each transcript, and any disagreements were resolved by discussion.

### Open Coding

We used an open coding method to understand the challenges that people with TBI faced while using Facebook. For the field notes and transcriptions, we conducted an open coding process in which codes were assigned to significant instances and references using Atlas.ti [50]. The first and second authors read the field notes and transcriptions repeatedly and coded them individually. Next, we compared each other’s codes and worked iteratively to find patterns and themes while resolving disagreements. To gain an in-depth understanding, we elaborated our coding schemes and analyzed relevant quotes to build rich descriptions and concrete examples of unique challenges faced by adults with TBI.

### Research Team and Stance

Our study team included researchers with extensive experience in rehabilitation services for people with TBI and computer scientists who specialize in creating social computing systems based on the think-aloud method. The combined expertise of these experts provided a solid foundation for understanding social media challenges faced by adults with TBI and developing design ideas for future tools to facilitate more active social media engagement.

## Results

### Overview

Cognitive test scores were obtained for 7 participants and are listed in Multimedia Appendix 1. Scores were unavailable for 1 participant as the tests were added to the protocol after they had completed the study. In the following sections participants are identified by number (eg, P 1). If participants self-identified as male or female, we used gender-specific pronouns in the results; otherwise, we used gender-neutral pronouns. Raw data are available in Multimedia Appendix 2, with participant transcripts organized based on the Meshi et al [51] subthemes. A description of each theme is provided in Multimedia Appendix 2 along with the number of times the code was assigned.

### A Priori Coding

#### Overview

Participants used all categories of codes in the Meshi et al [51] framework and also provided detailed feedback about the Facebook user interface. In some cases, participants *thought aloud* about what they were seeing on Facebook pages (eg, “There’s a cow playing fetch that’s pretty cool”) and in others they shared comments about how and when they used Facebook, prompted by what they were seeing (eg, “Sometimes when I am nosey I go on to see what I can see”). We included both types of comments in analysis, and in the following sections we describe themes that emerged.

#### Observe

The most common behavior from the Meshi et al [51] framework was to *observe* information broadcasted by others. Participants used Facebook to observe posts by paid sources, such as advertisements; news stories; socially shared videos, memes, and jokes; posts related to interests, such as musical groups and nature; and posts by others in their social networks, including friends, family, and employment-related contacts, which they used to keep up with what others “were up to.” Most participants were interested in observing content posted by friends and family to see “what’s going on” (P 3). Participants who were parents also used social media as a way of keeping up to date with the activities of their children. Some would scroll through to see if their children had posted anything new, while others would go through “the old pictures that [their children] have” (P 7).

Reasons for observing posts varied across participants. The sixth participant (P 6) only accessed Facebook to read things that other people posted, whereas P 5 accessed Facebook primarily for memes. P 3 indicated that she used Facebook because she was a curious person, and looking at people’s posts would allow her to form a better opinion of them.

P 2 stated that Facebook was a useful research tool for her to get a better sense of what kind of people her coworkers were. For example, if she saw that one of her coworkers was the type to share inspirational quotes like “live life or live long” (P 2), she would decide to limit her interaction with that coworker because this would indicate that they would be “sensitive to anything that I say” (P 2).

Participants P 1, P 2, and P 7 talked about being a member of a TBI group on Facebook. They reported that although they were not active on the pages, they did enjoy the content they would come across on those pages. Being able to share a common space with other members of their community was understood as a type of social interaction and one that kept them engaged and entertained.

#### Broadcast

Meshi et al [51] stated that individuals who are using social media either broadcast information in the form of a text, picture, video, link; or post something that is not in reference to themselves but rather an article or media content that they came across somewhere else. Participants in our study did both. As an example, P 2 both generated their own posts and also posted quotes and gifs from other web-based sources:

Every day I say hi everybody, happy Tuesday or happy Wednesday. This is what I cooked last night I did a chicken breast with some pepper and salsa

I do quotes every day. Like “if we take the mistake everyday of being grumpy or sour we are wasting today”

P 2

P 6 indicated that she used Facebook sometimes to share her political and religious views as well other facts about herself and her interest that she would like people to know:

I update my status about political topics and controversies. I also share my religious beliefs and views. I like that I can tell people something immediate happening in my life. On my profile you can see different movies and books I like. I have here some of my fav quotes

P 6

P 7 and P 8 both indicated that they posted pictures of their families going out on trips and engaging in different activities.

#### Compare

Meshi et al [51] stated that individuals tend to engage in social comparison by observing what others broadcast and receive feedback on and contrast that to their own user experience. Users also can compare variables such as number of friends, relationship status, and age. As an example, in this study P 3 made some comparisons while browsing through her Facebook feed:

I don’t like when people post their whole lives online. I find them to be self-absorbed like how important do you think you are?

P 3

#### Provide Feedback

Meshi et al [51] noted that social media can be used to provide social feedback to others. As this feedback is visible to the poster and often to the public, it contributes to the social comparison function of social media by providing *data* people use to compare themselves with others on dimensions such as popularity and likability. Participants described providing feedback to friends and others, including emojis (P 1: “Give that a hahaha emoji”), likes, and comments. P 5 used feedback to console a family member:

My sister-in-law’s sister had passed away today so she just posted a photo. I just want to put hugs on her photo for her.

P 5

P 4, P 5, and P 6 said they only made comments if posts were by family members, although they might “like” a post if they felt strongly.

#### Receive Feedback

According to Meshi et al [51], users receive feedback on posts they share through likes and comments. Receiving feedback is also another contribution to the social comparison function, as this feedback is also visible to the public. Participants described receiving feedback on content they shared on their social media. Feedback was in the form of emojis, comments, getting tagged, or direct messages. P 1 received feedback about a post he shared on his timeline: “Someone’s asking about the first tune.”

P 7 indicated that her children posted photos of her, tagged her in other photos, or sent her messages about things she had posted on her Facebook.

#### Patterns of Facebook Use

The a priori coding also revealed 2 dimensions that characterized participants’ Facebook use. The first dimension was whether they were active or passive about posting and self-disclosure on Facebook. Four of the participants were very active in posting, messaging, and disclosing themselves on Facebook. This included P 1, who stated the following:

I like to share my process about how I am doing things. It is more like I got up today, I brushed my teeth, so like, you know.

P 1

The remaining 4 participants used Facebook passively, mainly focusing on observing and consuming others’ posts. As P 7 replied when asked if he posted on his timeline, “No. I never have. It is not my thing.”

The second dimension was familiarity and fluency in using Facebook. Four participants described high confidence and fluency in using the variety of features and functions of Facebook, whereas the other 4 reported difficulties in learning and using Facebook. The distinction in fluency appeared to relate mainly to participant age. The first 4 participants were in their 20’s to 40’s and described themselves as relatively tech-savvy, and they were confident and capable of using digital devices and the Facebook Interface (eg, “Everything is easy to use. It is right there basically*”* (P 5). The other 4 participants were in their 50 to 60s and reported challenges in accessing and using Facebook (eg, “Sometimes I have a hard time learning things. There are all these other things that go with it that I don’t understand.” [P 3]).

### Open Coding

The open coding analysis revealed 6 major types of challenges reported by participants with TBI. Participants also provided suggestions for Facebook features and modifications that would support their access.

#### Difficulty With Language Production and Interpretation

Most participants expressed difficulties communicating on the web as part of the challenges they faced with social communication after their injury. As they lost contact with friends and acquaintances after injury, many of them had also lost confidence in communicating with others:

There are people I used to hang out with but I barely see them anymore...Either I scared them away or life in general I guess.

P 7

Some of the participants were unfamiliar with the communication norms and conventions of social media, and thus felt uncomfortable communicating with others on the web:

I wouldn’t do it online because I don’t like social media because I can’t control when to end the conversation.

P 2

Some participants reported challenges *reading* others’ emotions and intents. P 8 appeared to be uncertain about inferring the researcher’s thoughts and feelings, to determine appropriateness of a comment: “I want to ask you a lot but I don’t know if that’s appropriate.”

P 5 showed similar challenges:

But I can’t stop that when I meet people I want to tell them my life history. Now you don’t want to hear my life history, but I think you do.

P 5

Some participants showed awareness of their social cognition challenges, as in P 2’s comment about challenges conveying emotions:

When I am having text conversations, my sister said that my tone doesn’t carry well in my text. So when you are writing something, you have to be careful with your tone because people can’t read your face and tell that you are kidding or sarcastic and that just creates more problems. So that’s where I struggle with this.

P 2

P 6 likewise showed awareness of social cognition limitations in this comment:

I get self-conscious sometimes like what if what the person wrote wasn’t understood by me, what if they were trying to be funny and I didn’t understand the joke. I don’t wanna look stupid like I didn’t get what was said.

P 6

The absence of visual and nonverbal cues also led to some participants feeling less confident about the clarity and appropriateness of their messages when communicating on the web. Because of lacking confidence in social communication, participants stated that they became overly self-conscious about their communication skills and worried about their self-image on social media. For example, P 1 wanted to have a better spell checker so that he could compose better messages to improve his social image:

My spelling is bad. So it would be nice to have spellcheck to make me look a little bit better to my friends and stuff.

P 1

#### Information Overload

More than half of the participants reported they felt overwhelmed by the amount of information they received on Facebook and via the Facebook user interface:

Too much of that and too many videos keep going and going keeps going and going. It is overwhelming, sometimes too much.

P 6

The overwhelming feelings usually stemmed from the fact that the basic Facebook interface structure presented them with a comprehensive set of Facebook features. The interface was crowded and cluttered, and they wanted to “make it less crowded” (P 7) and customize it so that they could keep only the features they used most often on the screen.

Umm I would filter out...I wish I could control this. Like I don’t like the marketplace thing. I knew I am not interested in fundraiser so I would like to take them out of the side menu.

P 2

Also, some users felt lost because they were unable to quickly locate the posts or messages they wanted to keep and follow up. Whenever they saw the Facebook newsfeed showing the new list of posts that were automatically updated and algorithmically curated, they found it difficult to keep track of information. Participants wished for an “easier way of finding things” (P 6), so that they would not have to scroll or search indefinitely:

Like there are people I follow that posted a lot of stuff on here and now they are gone, what happened? Did they get taken off?

P 3

Furthermore, some participants found it particularly difficult to manage and catch up with notifications. This was due to some of them not knowing how to effectively manage the types and number of notifications they would receive, as illustrated in P 7’s account:

I didn’t know how to stop being notified of all the comments [from the post that I commented on earlier] Cuz it kept notifying me. I don’t comment any more.

P 7

As it was difficult for participants to keep track of all the notifications they received, they often forgot to respond to messages from their close friends:

My problem is that I contacted these people and then I forget [to respond to them]. Then 5 months later I am like I haven’t done anything about that.

P 3

#### Emotional Contagion and Emotion Overload

A few participants expressed negativity toward the posts they saw, such as having a “short tolerance for people” so they “ignore them” most of the time (P 4). A few participants also reported that they were easily influenced and triggered by emotions and topics in posts. For example, P 1 stated that he was very triggered by every topic he saw in the post, and he expressed all of his thoughts and feelings about each post. Similarly, P 7 reported that the posts from TBI survivor groups made her feel very sad: “It’s nice to see things that are related to me but sometimes it makes me sad.”

As a way to regulate their triggering feelings and negative thoughts while browsing their newsfeeds, P 6 suggested a mood-based filter that would curate the posts based on their mood:

I wanna see things based on my mood. If I’m happy, I don’t wanna see posts about sad things. Don’t make me sad.

P 6

#### Insufficient Guidance to Use Facebook

Participants who were in their 50s and 60s said they did not feel they could use Facebook to its full potential because of insufficient guidance resources. In some cases, their children helped them to set up their account (eg, P 3), but they still felt that they did not have much knowledge or guidance to effectively use Facebook:

My kids put this (Facebook profile) up for me.

P 3

I wanna see pictures my kids post. They should send them to me [directly], but they share them here. My kids tag me in their photos or they message me. I tell them to call me instead.

P 6

Many participants found it particularly challenging to locate and retrieve information. For example, as mentioned earlier, many participants found it particularly difficult to retrieve posts they wanted to keep:

There was this one thing I saw. I wish I could find it. I always lose things I find interesting. My daughter says I can save them but that’s hard anyway.

P 7

Although Facebook offers a feature to save and retrieve posts users want to keep (the *Save* feature), many participants were not aware of it or how to use it. Particularly, they felt overwhelmed by the constantly changing interface of Facebook. When the Facebook layout they were familiar with was changed or removed, they felt confused. For example, P 6 mentioned that having to learn everything all over again made him not want to stay on Facebook:

It looks different every few years, which bothers me too. I get used to it then it changes, which makes me not want to use it because I have to learn again. I would stop changing it.

P 6

#### Internet Scams and Fraud

A few participants expressed concern about potential internet scams targeting people with brain injuries. P 2, who was involved in the local TBI community, stated that many TBI survivors were vulnerable to internet scams:

The other concern I have about this, especially for people with brain injury. When it comes to this, there is potential for fraud for people to be taken advantage of...I see a lot of fraud people take advantage of romantically. They can scam and rip people off.

P 2

Some of the participants were aware of potential scams, so they did not respond to people outside of their close social network:

I don’t talk to strangers much. I get random messages, but I don’t respond unless I like know them.

P 5

To reduce the potential risk of internet fraud, P 2 recommended limiting Facebook friends to members with something in common, such as belonging to the same TBI support group or enjoying activities together.

#### General Accessibility Concerns

Participants also raised the issue of general web accessibility. For example, a few participants found that on Facebook “the font is so small and very faint” (P 2), which could be a problem because “a lot of people with brain injury have trouble seeing” (P 1).

Another user stated that it was difficult for her to understand how she could stop being notified when she posts a comment on a photo. She said that, “other people started commenting [on the post] and I didn’t know how to stop being notified of all the comments...so yeah, I don’t comment anymore” (P 3). The same user identified problems saving posts that she would like to access later, saying, “my daughter says I can save them, but that’s hard” (P 3).

## Discussion

### Principal Findings

We invited 8 adults with TBI to describe their thoughts and actions as they navigated their Facebook accounts. We chose the *think-aloud* method to better understand how Facebook functioned for these users, as well as their challenges and barriers, and to avoid limitations of previous studies that relied on retrospective recall and self-reflection. Participants’ comments showed the 5 key social media behaviors described by Meshi et al [51], and extended beyond those, capturing important Facebook functions and barriers to use. In the following sections, we discuss both sets of results and suggest Facebook features and modifications that could address barriers identified by adults with TBI in this study.

Meshi et al [51] proposed that adults use social media to broadcast information, receive feedback, observe others, provide feedback, and compare themselves to others. These behaviors were based on how social media is used to satisfy the basic social needs of healthy individuals, to guide research in social neuroscience. We found the framework equally useful for understanding Facebook use among adults with TBI. Participants in our study showed the same social behavioral motivations as uninjured adults, and both the content and structure of their comments revealed barriers to using social media to fulfill basic social functions.

Open coding of participants’ comments revealed unique challenges that adults with TBI faced while using Facebook. These challenges included structural barriers, such as distracting visual content and frequent updates that change the user interface; content barriers, such as information that triggered negative feelings; learning barriers, such as lack of accessible guides to Facebook use; and safety concerns related to the risk of internet fraud and exploitation. Participants’ comments also revealed their strengths and challenges in cognitive functions needed for successful social media use, including evidence of impairments in social cognition, which have been extensively documented in the TBI literature [17,21,52-54]. Participants’ comments also revealed strengths in these areas, including self-reflection on how their own social media posts could be interpreted by others. A few participants were using Facebook as a way of “keeping up with the news” (P 6). This could be a concern, given that news excerpts on Facebook are typically trimmed and not always accurate or reliable sources of news.

### Comparison With Prior Work

Our results aligned with findings from previous studies on social media use in adults with TBI [3,29,32]. Adults with TBI interviewed by Brunner et al [32] reported similar feelings of being overwhelmed and cognitively fatigued by the demands of using social media and confused by technological variations across platforms. About 1 in 4 adults with TBI surveyed by Baker-Sparr et al [29] reported difficulty using social media because of their TBI-related challenges, including memory problems and general technical difficulties with site functions. Cognitive challenges may also discourage potential users from trying Facebook, as was the case with nonusers with TBI in a Facebook survey by Tsaousides et al [3]. Safety concerns were identified by participants in a few studies and were the main focus of clinicians interviewed by Brunner et al [9].

### Future Directions

In addition to identifying challenges, participants recommended features that could help them to use Facebook more effectively. Table 1 summarizes the main suggestions from participants, along with our own ideas for features.

**Table 1:** Challenges for adults with TBI using Facebook, and features suggested by the authors to improve access.

Challenges	Feature suggestions
Difficulty with language production and interpretation	Visual aids (emojis and appended images) Spelling and grammar check functionality Aids to analyze and monitor tone and meanings
Information overload	Simpler, customizable interface Setting that selects types of notifications Cues to follow up with messages An easier way to locate and search information
Emotional contagion and emotion overload	Filter to hide posts expressing strong negative emotions Mood-based feed curation
Insufficient guidance to use Facebook	Way to revert back to the old interface Interactive guide
Internet scams and fraud	Friend request filter to limit friends to someone who shares a common connection or background
General accessibility concerns	Larger text font and user interface elements (eg, buttons)

### Supporting Language Comprehension and Production

To provide adults with TBI with resources to interpret and express the meaning and tone of text messages, future systems might encourage users to include visual cues such as images and emojis. A spelling and grammar checker that is universal across social media sites could also assist users in composing messages with more confidence. System developers can create new interfaces that automatically analyze and provide information about sentiment, tone, and emotion in texts and images using natural language and image-processing methods. These tools would support comprehension and expression of literal meaning, as well as meaning that requires mentalizing (eg, understanding others’ emotions and responding appropriately).

### Reducing Information Overload

Users with TBI could benefit from a simplified and customizable interface that keeps only the features that users want on the screen and allows users to control the types and number of notifications they receive. As participants reported that excessive notifications often made it difficult to follow up with meaningful relationships, system developers could also provide mechanisms to prioritize certain types of notifications and remind users to reply to high-priority notifications. Such options, however, must be presented in ways that do not overwhelm users, for example, in the forms of presets from which users can select. In addition, it would be beneficial to have simpler and more intuitive ways to store and retrieve fast-fading information, as many participants could not easily locate and keep track of the information they want to retrieve.

### Minimizing Emotional Contagion and Emotion Overload

Social media sites may provide users with a customizable filter that users could set to block emotionally triggering topics and people, so that they do not have such content on their feeds. Furthermore, a mood-based filter using sentiment analysis models could curate posts based on the user’s mood.

### Providing Accessible Guidance for Using Facebook

As many participants felt that there was little guidance and information on Facebook, there should be an easily accessible and universal guide to Facebook. As some participants expressed lack of confidence to learn unfamiliar features, it would be useful to provide materials or websites that would guide both users and others in their lives on how to use Facebook. Users commented on challenges with the interface changing with updates, it would be helpful to have an option to revert back to the old version of the interface, as that would reduce confusion and frustration caused by changes to the layout and features.

### Protecting Users From Internet Scams

One participant suggested a filtering feature to allow users to select “Facebook friends” who have something in common, such as belonging to the same TBI support group or participating in the same activities. Such a screening process could reduce the potential risk of internet scams. In a qualitative study of rehabilitation professionals’ views on social media use after TBI, Brunner et al [9] found that professionals often viewed their roles as “gatekeepers,” to protect individuals with TBI from exploitation and other harms associated with social media use. If users with TBI had more control over their networks, they could have more autonomy in choosing friends rather having professionals in the gatekeeper role. Facebook allows users to control who they choose to “friend,” but it is not possible to choose a subgroup of friends with whom to share a post. A friend-subgroup-selection feature could be useful, although we acknowledge that it would require a multistep routine that could be challenging for many adults with TBI.

### Improving Overall Accessibility

As TBI affects visual processing abilities, future social media sites will need to make it easier for users to resize font size and user interface elements such as buttons on their websites.

### Limitations

The first limitation of this study was the small sample size, with participants from the same geographic region. These limitations were unanticipated consequences of pandemic restrictions in 2020. Despite the small and relatively homogeneous sample, participants generated a range of Facebook uses and recommendations, but information from a larger sample would be informative.

A second limitation was the challenge for some participants in following the think-aloud protocol. This group of participants had impairments in speed of processing, verbal learning, and shifting between tasks. That cognitive profile represents the target population for whom we aim to improve social media access, which was a strength of the study, but use of the think-aloud method in an unstructured task like browsing might have been overly challenging. Participants also sometimes initiated conversation with the researcher on the content that they saw on Facebook rather than discussing their Facebook use. The study could have been done with the researcher outside the room, but that would be atypical for a think-aloud study and would have its own set of challenges, for example, inability to cue participants if they stopped commenting.

A third limitation of the study was that we observed users with TBI in a single session and on a device provided by the researcher, which likely did not reflect the users’ experience more broadly. Participants might have used the platform differently if they were using their iPad, tablet, or mobile phone, and the appearance and functions of Facebook differ across different devices. Although this study provided useful preliminary information, future research should include extended use of the participants’ preferred platform on their preferred device

### Conclusions

Results of this study provided insights into the benefits and challenges of Facebook use for adults with TBI. A key finding was that participants in this study used Facebook for the same functions as typical adults, which suggests that Facebook and other social media might help reduce the social isolation and loneliness often reported by people with TBI. Although participants’ intentions were like those of typical adults, their experiences were not: participants encountered significant barriers, including both features of Facebook that could be challenging to anyone, such as being bothered by advertising, and also barriers specifically due to their TBI-related cognitive impairments, such as challenges in inferring others’ thoughts and feelings and expressing their own feelings and intents. As a result, Facebook use was often a frustrating experience that increased rather than decreased social isolation.

While barriers identified here were similar to those reported in previous studies of social media use after TBI [29,32,33], the think-aloud method yielded unique information about specific features of Facebook that posed challenges for users with TBI. The study findings in turn suggested modifications and technological aids that could help people with TBI succeed in the web-based social world. If supported by future studies in larger groups, these modifications, could support people with TBI in being part of web-based social and community life. As increasing social media use also can be a target of rehabilitation, the type of clinician training proposed by Brunner et al [22] also would be important, as clinicians might not have specific skills in how to support social media access for individuals with TBI.

## Appendix

Multimedia Appendix 1 Participant cognitive test scores.

Multimedia Appendix 2 Participant transcripts.

## Abbreviations

TBI: traumatic brain injury

## Data Availability

Raw data are included as Multimedia Appendix 1.
