# Supplementary material for: Facebook Experiences of Users With Traumatic Brain Injury: A Think-Aloud Study
Source: JMIR Rehabil Assist Technol. 2022 Dec 16;9(4):e39984. doi: 10.2196/39984 (PMC9804090; doi:10.2196/39984)
Supplement: Multimedia Appendix 1 [file rehab_v9i4e39984_app1.docx]

**Appendix 1. Cognitive Test Scores**

*Cognitive Test Scores for seven participants with TBI. Data are median scores with range in parentheses. Data were not available for an eighth participant who was a male age 25-35 years.*

|  | *M* | SD |
| --- | --- | --- |
| Trails A | -.5 | 1.29 |
| Trails B | -1.82 | 3.40 |
| WAIS-PSI | 75.29 | 13.82 |
| CVLT First Trial | -.78 | 0.86 |
| CVLT Immediate | -.93 | 1.43 |
| CVLT Short Delayed | -1.64 | 1.46 |
| CVLT Long Delayed | -1.93 | 1.27 |

*Note.* TBI = Traumatic Brain Injury. *M* = mean, SD = standard deviation, CVLT = California Verbal Learning Test [1], Trails A: Trail making Test Part A, Trail B: Trail making Test Part B [2], WAIS PSI = Wechsler Adult Intelligence Scale [3] Processing Speed Index. Trails A and B scores are z-scores; CVLT, WAIS, and PSI scores are scaled scores with a mean of 100 and standard deviation of 15 in uninjured adults.

**References**

1. Delis DC, Kramer JH, Kaplan E, Ober BA. California Verbal Learning Test - Adult version (CVLT-II). Second ed. Austin, TX: The Psychological Corporation; 2000.

2. Tombaugh TN. Trail Making Test A and B: normative data stratified by age and education. Arch Clin Neuropsychol. 2004 Mar;19(2):203-14. PMID: 15010086. doi: 10.1016/S0887-6177(03)00039-8.

3. Wechsler D. Wechsler Adult Intelligence Scale. Fourth ed. San Antonio, TX: Pearson; 2008.
