# Supplementary material for: Facebook Experiences of Users With Traumatic Brain Injury: A Think-Aloud Study
Source: JMIR Rehabil Assist Technol. 2022 Dec 16;9(4):e39984. doi: 10.2196/39984 (PMC9804090; doi:10.2196/39984)
Supplement: Multimedia Appendix 2 [file rehab_v9i4e39984_app2.docx]

**Appendix 2. Participant transcripts**

The following table is a transcript of all participants dialogue during the think-aloud task. It has been broken down into themes based on the Meshi et al.Code. Under the heading “Meshi et al.Code” we have labeled the theme and the number of times reference to that particular theme has been made. Under the heading “example” we have provided a transcript of all the participants who made comments that could be categorized under the specified theme.

| Meshi et al. Code | ID | Example |
| --- | --- | --- |
| Broadcast  Total codes: 31  User is broadcasting information (e.g., text, pictures, links, videos, etc.). This can either be personal information or content that is not related to them in any way | P1 | alright go / um / I'm talking to my mom about I vehicle I want to buy / and I was researching / um / how to get temporary plates / so I'm telling her that I need/ so maybe she can get the temp temporarily plates / since it's such short notice to get insurance plate |
|  |  | What’s going on here? / I love these / like Amish ladies / or Muslim ladies / or something / dancing to drum and base/ That’s funny / I just posted a thing of a bunch of nuns doing that/ it must’ve been like two hundred of them |
|  |  | is this suppose to be a service dog / We had a situation where we had two dogs/ But he actually is a very great service dog / I think she kept him on YouTube / We use to bring like pop bottles / or anything to try and play fetch/ in the lab/ a really nice lab / I'm commenting on it / Sharing that I remember playing fetch with it / with just about anything |
|  | P2 | I use it for my profile |
|  |  | For everyday I do a video /This is this morning |
|  |  | Everyday I say hi everybody, happy Tuesday, or happy Wednesday |
|  |  | This is what I cooked last night / I did a chicken breast with some pepper and salsa |
|  |  | I do quote every day / Like "if we take the mistake everyday of being grumpy or sour we are wasting today" |
|  |  | My status today is: so excited to go to McMaster today. |
|  |  | Yeah, and the picture / I took a picture of myself saying "hey, I am on the road and ready to go" / I like to keep people informed |
|  |  | that’s my tattoo that I have. It is the year that I had my accident |
|  |  | I like to share my process / How I am doing things / It is more like I got up today / I brushed my teeth / so like / you know |
|  | P6 | To keep in touch with friends. Post some of my photos and things that I like |
|  |  | I don't use it to update everyone on my relationship status |
|  |  | I post pictures of political things I support |
|  |  | or I update my status about political topics and controversies |
|  |  | I also share my religious beliefs and views |
|  |  | I like that I can tell people something immediate happening in my life |
|  |  | On my profile you can see different movies and books I like |
|  |  | it gives people insight into the kind of person I am |
|  |  | I've made my Facebook page public so that my stuff can be seen by everyone |
|  |  | I have here some of my fav quotes |
|  | P7 | I posted pictures of my family / going out places |
|  |  | I would be active on my birthday/ but I wouldn’t post too much / I would share news stuff / random YouTube links |
|  | P8 | normal, to post things / I post lots of albums/ family trips |
|  |  | here is a picture of my dog / its my second dog actually / I had another dog that died/ Facebook keeps all the photos for me |
|  |  | yeah, I do / see / these are some pictures / some of the videos I share/ I like to think people want to see/ what I have to share |
|  |  | sometimes/ sometimes I just post it because/ later I want to go back and see it/ its easier to find when its on/ my own/ profile |
| Receive Feedback  Total Codes: 5  Participants are getting feedback on content and posts they have shared (e.g., a user shares a photo and others begin commenting on it directly or push like or react) | P1 | Someone’s asking about the first tune. |
|  | P5 | Yeah. But If it's just stupidness, I just ignore them. I have a short tolerance for people. And umm and I just ignore you |
|  | P7 | My kids tag me in their photos / take funny pictures of me sometimes / or they message me |
|  | P8 | oh yeah/ I like things / I don’t like when I post something/ and everyone ignores it/ so I don’t do that to people/ even if I’m the only one to like it/ I will like it |
| Observe  Total Codes: 88  The participant is observing information that has been broadcasted by others (e,g., observing old posts, watching videos on news feed etc.) | P1 | Okay / Just checking because I had notifications from this guy / it's my sister's boyfriend / We’re playing pool on Facebook messenger/I'm kind of I don't know my mom got me into it |
|  |  | I’m just going to scroll my feed / I’ve checked all my messages / Just scrolling / Find something maybe interesting / |
|  |  | What’s this? / Bad parenting / There’s no volume or?/ Got it / Actually / which f key is it to make more volume? / More volume / Oh there it is / Normally there with the keyboard |
|  |  | This kid’s stuck in the machine with the claw that gets the toys/ I missed something here / oh / they’re basically just laughing at her you don’t see how she gets in/ there’s two of them / I’ll machine them out / Oh my god / they’re (inaudible) the toy machine it doesn't say / She’s trying to pull her through / She got / they got the kid though/ That’s pretty funny |
|  |  | It’s my lovely aunt / What’s going on here? / Oh, that’s pretty /I don’t know where that is / She must be on vacation somewhere / There’s a lot of comments / ope nope / just five/ Something in French /Something else in French / I’m going to ask her where that is |
|  |  | Oh man / it's like ridiculous/ Like don’t throw your baby / Don’t wash him with a hose / Don’t put him in a hamster cage / Don’t expose to outlets with the wires / This was posted by my buddy Myles / How to check for poop/ Look /Or stick your hand down there / Look / Or stick your hand down there / That’s funny / Give that a hahaha emoji |
|  |  | There’s a cow playing fetch / That’s pretty cool / I feel bad for cows / They’re smart, but beef is delicious |
|  |  | It’s a movie trailer/ Oh it’s Netflix / Maybe a tv show / Emotionally triggering video/ Video of a movie review I don't know |
|  |  | Hmm / Seems like a controversial / kind of interesting post only true men of peace have the courage to tell the painful truth in order to avert the bigger conflict / the Dalai lama / Europe belongs to the Europeans / The Dalai lama spoke at a conference in Malmo photo / And he comments / perhaps the world’s most famous refugee / He longs to be able to return to his own country / |
|  |  | While he is grateful for the help he had been given in India / he knows he doesn’t belong there / He is a courageous and honest spiritual / and we should be thankful for his words /because we continue to be failed by our spiritual leaders in the West |
|  |  | It’s a black and white kid / And they got the same hair cut / to trick their teacher/ So it goes from far right to far left |
|  |  | This is my baby / Oh that's cute |
|  |  | Some artist/ fast forward through this to see what he does / oh cool he does glass wow he made like three paintings done on glass really quick |
|  |  | some music / some festivals and concerts and stuff / someone supporting cannabis / this guy has skin cancer / some medical marijuana group / I’m guessing it helps him / helps him out this dog / service dog educational |
|  |  | this is um making fun of people that don't like prong collars / my girlfriend’s a dog trainer so she like complains about them all the time/ people misuse them / like / they wear them wrong / and are like / oh they're so bad/ like / they keep them on their dogs all the time / instead of just when they're walking them/ or they’ll make them way too loose / they’re supposed to be tight up by the neck / under their jaw/ but yeah actually / it's actually hurts / can hurt the dog more when they pull on a leash / because it's constant pressure on their spine/ whereas they pull / get a correction / and are walking good / there's no constant pressure on them / like a leash |
|  |  | this seems funny/ what the heck is happening mate/ that’s funny scrolled down, and it kept playing |
|  |  | Trudy’s playing uh pool and bowling |
|  |  | Hmm something about poker / Meh |
|  |  | Someone’s asking about the first tune? |
|  |  | That’d be cool birthday party DJ |
|  |  | Oh, it's um a memory from twenty ten / That’s pretty funny / Takes some balls |
|  |  | Some cop’s helping a turtle / Save the turtles / I’ll like that / I like it when cops do things like that / Or help old ladies cross the road / Or like stop for the whole traffic / I've never seen that / but on Facebook I have |
|  |  | This is a post by my mom / businesses refuse gypsies |
|  |  | Chadgie gadgie / Boo hoo / It’s crazy though / They’ve been there two thousand years / Is that what he said?/ Gypsies are everywhere man/ A thousand years/ As long as you’re not deviant and stealing / I don't care/ That’s probably why they’re / people don't like them / because in the past they'd be / sketchy/ It’s not because they’re traveling people/ It’s because they’re sketchy people / It’s almost like being anonymous/ Because they can just travel around / Like screw people in this area / and go to the next one/ But I don’t think it’s really like that these days / So / it sucks that people are still ban / still banning them from stores and shit/ it’s probably just / you know old people/ that will phase out |
|  |  | What’s going on here? / I love these / like Amish ladies / or Muslim ladies / or something / dancing to drum and base/ That’s funny / I just posted a thing of a bunch of nuns doing that/ it must’ve been like two hundred of them |
|  |  | Bruce trail/ who's this/ that's cool |
|  |  | that cool / elbow flexing where you fold down into the video/ huh |
|  |  | wow that’s packed/ what’s going on/ Russian FM Lavrov holds final press conference on sidelines/ Oh my god / this is an hour long |
|  |  | The four candles/ Ehh/ She’s kind of sappy though/ who's this guy/ He's probably stupid |
|  |  | His girlfriend is going as medusa / Greek mythology it is |
|  |  | Is that a commercial in between the video/ Ooooh / that drives me nuts  ahh / what / what / there's ants that are moving a bunch of petals / around a dead bumble bee/ Just like why they would do that/ but It looks really beautiful/ I guess you can interpret it as a savage bumble bee |
|  |  | There is a cat playing in the mirror |
|  |  | What’s going on over here/Poor people/ Who's whistle / oh it's more than just whistling /It’s a language/ What! / no way Food is ready/ Okay we're coming / Woah no way/ It says Turkish villages whistle / to communicate/ It's like some covert shit/ Cool population that speaks whistles/ And their kids are taught it / Wow scary |
|  |  | That's me/ Someone made me their cover picture / Oh man/ When was this picture taken / I don’t know/ I was pretty young though/ hmm / that’s maybe / like six years ago /I think we went to / no five years ago / we went to a golf course |
|  |  | South Park/ What do you have to say/ Those guys are funny / the people who make South Park/ This is making fun of school shootings/ it's saying / it's like the same as a/ math test / obviously it's not / but/ South Park / She works at a green house / That's very funny |
|  |  | Is this suppose to be a service dog / We had a situation where we had two dogs/ But he actually is a very great service dog / I think she kept him on YouTube / We use to bring like pop bottles / or anything to try and play fetch/ in the lab/ a really nice lab / I'm commenting on it / Sharing that I remember playing fetch with it / with just about anything |
|  |  | Cool chick / She's um / in vet school or something |
|  |  | Oh, harry potter stuff / lets see it/ Every time I watch a video I wish I could speed it up / Awe poor Cy / he turns out to be okay in the end/ Good old dumbledwarf/ I always believed in Cyrus |
|  |  | I hate heights / This is some sky bridge / Oh it’s a crane/ Make a pool by yourself |
|  | P2 | I check on my family posts |
|  |  | This group is called online tb survivors |
|  |  | This picture is two years ago. / They are all old pictures / this one is the oldest |
|  | P6 | I am on different groups and pages. I am not active on them, but like the things they post |
|  |  | sometimes you have people saying really dumb things |
|  |  | I prefer to read them |
|  |  | The main thing I like using it for is memes |
|  | P4 | What do I do? Just scan through it and see what my kids put on there and that’s it. |
|  |  | Yeah, I scroll through everything |
|  |  | My daughter’s best friend |
|  |  | I come out here and check what’s going on |
|  |  | Like there is bands I follow, and groups I follow sometimes. |
|  |  | Sometimes there is good videos on here / interesting things |
|  |  | my son does but I go through it to see what’s on there |
|  |  | Well, here are some bands that I follow. |
|  |  | There is me. Thats (participants name). |
|  |  | There is a page of city |
|  | P5 | Sometimes when I am nosey, I go on to see what I can see |
|  |  | Scroll through newsfeed page. |
|  |  | My sisters-in-law sister had passed away today so she just posted a photo. I just want to put hugs on her photo for her. |
|  | P7 | I use Facebook / but only to read things that other people post  but even then, I don’t talk to them / about what they posted  sometimes there is random videos like this / and that’s my way of keeping up with the news |
|  |  | ha-ha / he's funny |
|  |  | ha-ha / wow/ so much / who sits there to write though |
|  |  | I watch and move on to the next thing |
|  |  | I want to see pictures my kids post |
|  |  | ha-ha these are some of their friends |
|  |  | this is stuff I’m okay to see |
|  |  | maybe less of those funny things / meme things they called / ha-ha |
|  | P8 | My husband has me join some brain injury pages / he’s good that way/ I sometimes go on there/ its nice to see the things that are related to me/ but sometimes it makes me sad/ they have these stories sometimes/ you know |
|  |  | click all the red things / the notifications / the messages/ I don’t get much of either/ usually there is no red thing/ but I just scroll down / till I see a post that’s interesting/ then I watch it or something/ sometimes I will go to my kids’ page/ look to see if they put anything new / or go through the old pictures they have |
|  |  | I look at my daughter’s boyfriend page/ he’s nice to me/ I always like his stuff / I see what he’s up to |
|  |  | I have before / like there was this one thing I saw/ wish I could find it/ I always lose things I find interesting / my daughter says I can save them/ but that’s hard / anyway I don’t even know / what the post was about/ but I talked on there/ then other people started commenting/ and I didn’t know / how to stop being notified/ of all the comments/ because it kept notifying / so yeah, I don’t comment any more |
|  | P3 | No, I tended to just have it to look at what people wrote |
|  |  | If I thought, to me this is kind of dumb / so I wouldn't comment on it, just because / I would just skim it |
|  |  | Sometimes I feel obligated to make a comment / so I just don’t |
|  |  | And also, I am kind of curious / so I would look at people’s posts / then I was able to form an opinion more of them / |
|  |  | because, especially for co-workers / I would look and be like if they have / you know those people who post inspiration quotes and stuff and whatever / "live life or live long" or whatever / I would be like okay if they think this way, I am going to avoid them / because they are going to be sensitive to anything I say |
|  |  | I would follow a lot of planet earth and nature things / this is one called "birds of the earth" / things that are calming |
|  |  | and I follow the Ontario brain injury services |
|  |  | I also follow my employer |
|  |  | like I had a friend who kept posting everything / and she got a support dog / and so I stopped |
|  |  | But now she has a baby |
|  |  | and I follow certain charity like Hamilton fire fighters’ charity / because of my work relationship |
| Provide Feedback  Total Codes: 23  Participants are providing feedback on the posts of others (e.g., like a picture of a friend or post a comment on what someone else had posted) | P1 | Heading back to the homepage now / Going to like that post |
|  |  | Oh man / it's like ridiculous/ Like don’t throw your baby / Don’t wash him with a hose / Don’t put him in a hamster cage / Don’t expose to outlets with the wires / This was posted by my buddy Myles / How to check for poop/ Look /Or stick your hand down there / Look / Or stick your hand down there / That’s funny / Give that a hahaha emoji |
|  |  | This is kind of personal about me but / I agree that we have way too much immigration/ So, I’m going to like that post |
|  |  | I'm going to tag someone/ She'd enjoy this / Just looking for something interesting/ The same south park video/ I like that |
|  |  | Is this suppose to be a service dog / We had a situation where we had two dogs/ But he actually is a very great service dog / I think she kept him on YouTube / We use to bring like pop bottles / or anything to try and play fetch/ in the lab/ a really nice lab / I'm commenting on it / Sharing that I remember playing fetch with it / with just about anything |
|  | P6 | maybe, depends on what it is/ if I feel really strongly, I might push like |
|  |  | I am not very likely to like the post of random people or comment though |
|  |  | Unless they are my friends and family |
|  |  | I often like their posts and comment |
|  | P4 | I will send comments once in a while if there is something |
|  |  | Like he is my nephew so I will send him a comment. It is usually people that I know |
|  | P5 | Yeah, and If I see someone yapping, I comment on that too |
|  |  | My sisters-in-law sister had passed away today so she just posted a photo. I just want to put hugs on her photo for her. |
|  | P7 | no, I'm not like that. I see some posts have 3,000 comments |
|  |  | sometimes I will be tempted / but there are so many comments / I even lose the one I want to reply on |
|  |  | If its someone I know / I would push like probably / maybe comment if its my family |
|  |  | I want them to know I care |
|  | P8 | oh yeah/ I like things / I don’t like when I post something/ and everyone ignores it/ so I don’t do that to people/ even if I’m the only one to like it/ I will like it  I talk a lot / I think only the people who know me/ want to talk with me/ no I don’t want to bother strangers/ or people who haven’t / you know/ spoken to me in a long time /I wish I was one of those people/ I am social but shy / does that make sense/ I know its funny to be both / like you / I want to ask you a lot but I don’t know if that’s appropriate |
|  |  | I have before / like there was this one thing I saw/ wish I could find it/ I always lose things I find interesting / my daughter says I can save them/ but that’s hard / anyway I don’t even know / what the post was about/ but I talked on there/ then other people started commenting/ and I didn’t know / how to stop being notified/ of all the comments/ because it kept notifying / so yeah, I don’t comment any more |
|  | P3 | yes, I would like it / but I wouldn’t make a comment |
|  |  | So, I will say to her hey, do you know what your schedule looks like? / Can you schedule me in |
| Compare  Total Codes: 2  Participants make remarks regarding how they think others perceive them or the way they perceive others. | P3 | I don’t like when people post their whole lives online/ I find them to be self absorbed / like how important do you think you are? |
|  | P7 | also, I get self conscious sometimes / like what if what the person wrote wasn’t understood by me / what if they were trying to be funny / and I didn’t understand the joke so yeah / I don’t want to look stupid like I didn’t get what was said |
